# Pediatric Tele–Home Care Compared to Usual Care: Cost-Minimization Analysis

**DOI:** 10.2196/31628

**Published:** 2022-01-20

**Authors:** Cristina Adroher Mas, Candela Esposito Català, Astrid Batlle Boada, Ricard Casadevall Llandrich, Marta Millet Elizalde, Juan José García García, Manel del Castillo Rey, Francesc García Cuyàs, Miquel Pons Serra, Francesc López Seguí

**Affiliations:** 1 Sant Joan de Déu Hospital Catalan Ministry of Health Esplugues de Llobregat Spain; 2 Centre de Recerca en Economia i Salut Pompeu Fabra University Barcelona Spain

**Keywords:** cost analysis, pediatric tele–home care, home care service, health economics, telehealth, economic evaluation, telemedicine, pediatrics

## Abstract

**Background:**

Although home hospitalization has been a well-known and widespread practice for some time in the adult population, it has not been the same case in the pediatric setting. Simultaneously, telemedicine tools are a facilitator of the change in the health care model, which is increasingly focused on home care. In a pioneering way in Spain, the in-home hospitalization program of the Hospital Sant Joan de Déu in Barcelona allows the child to be in their home environment at the time they are being monitored and clinically followed by the professionals. Besides being the preferred option for families, previous experience suggests that pediatric home hospitalization reduces costs, primarily thanks to savings on the structural cost of the stay.

**Objective:**

The aim of this study is to compare the average cost of a discharge by tele–home care with the usual care and to analyze the main drivers of the differential costs of both care models.

**Methods:**

A cost-minimization analysis is conducted under a hospital’s perspective, based on observational data, and estimated retrospectively. A historical control group of similar patients in terms of clinical casuistry to children hospitalized at home was used for comparison.

**Results:**

A 24-hour stay at the hospital costs US $574.19, while the in-home hospitalization costs US $301.71 per day, representing a saving of almost half (48%) of the cost compared to usual care. The main saving drivers were the personnel costs (US $102.83/US $284.53, 35.5% of the total), intermediate noncare costs (US $6.09/US $284.53, 33.17%), and structural costs (US $55.16/US $284.53, 19.04%). Home hospitalization involves a total stay 27.61% longer, but at almost half the daily cost, and thus represents a saving of US $176.70 (9.01%) per 24-hour stay.

**Conclusions:**

The cost analysis conducted under a hospital perspective shows that pediatric tele–home care is 9% cheaper compared to regular hospital care. These results motivate the most widespread implementation of the service from the point of view of economic efficiency, adding to previous experiences that suggest that it is also preferable from the perspective of user satisfaction.

## Introduction

Although home hospitalization in adult hospitals is widespread and well known, and has been well studied from an economic perspective [1-3], it is not the same case in the pediatric environment, with a few exceptions [4,5]. Home is a child’s natural environment. The European Association for Children in Hospital Charter establishes that a child should only be admitted to the hospital if absolutely necessary and must be discharged as soon as possible [6]. At the same time, currently available telemedicine tools allow real-time monitoring of a patient’s clinical status and regular follow-up with families [7]. In this sense, technology is a facilitator of the change in the care model, and it is increasingly oriented toward home care [8,9].

The Sant Joan de Déu Hospital in Barcelona is a third-level university hospital located in Catalonia, Spain, specializing in the fields of pediatrics, gynecology, and obstetrics. It is a privately owned hospital that operates as part of the public health system. It sees approximately 26,000 discharges annually, with around 250,000 outpatient consultations; 15,000 surgical interventions; and 120,000 emergencies. This health center plays a double role in the Catalan health system: on the one hand it is the reference hospital for the population of the nearest geographical area; on the other, it is a high-complexity reference center at a Catalan, Spanish, and international level. Consequently, the population treated in the hospital presents pathologies of both low and high complexity. The program “SJD a Casa” (SJD At Home) of the Sant Joan de Déu Hospital, a pioneering initiative in Spain, was born in response to this need, and it allows the child to be monitored in their home environment while being followed clinically by the hospital professionals. It is an alternative for stable patients who require hospital treatment but not its infrastructure. Home hospitalization empowers the patient and their families, who can get involved in the direct care of the child, increasing their comfort and promoting family-centered care. Prior studies show that home hospitalization is safe [10] and that clinical effectiveness is not significantly different to conventional hospitalization, even for pediatric patients [11]. Furthermore, prior reporting states that experiences are positive [12-19]. After the success of the pilot program, with families preferring home hospitalization in 94% (61/65) of cases [20], “SJD a Casa” started operating in an ordinary way on November 1, 2019.

In a situation where the capacity to expand hospital beds is limited by the lack of space, especially in an urban context, this model of care frees up space by increasing the capacity to care for highly complex patients [21]. Previous experience suggests that pediatric home hospitalization reduces costs, relative to usual care, especially because of the effect of savings on the structural cost of the stay, which more than offsets the costs of possible readmission [22]. In addition, in a pandemic state, minimizing contact with users may be especially appropriate to prevent outpatient infections [23]. In this context, the aim of this study is to perform a cost-minimization analysis from a hospital perspective.

## Methods

### Study Design

A cost-minimization analysis was performed based on an observational study, including both direct and indirect costs. The analysis followed the Consolidated Health Economic Evaluation Reporting Standards (CHEERS) [24,25]. The study spans from November 1, 2019, to June 30, 2020; it assesses the time horizon from admission to discharge, and it has been conducted under a hospital perspective. No discount rate was used. Unidentified clinical and sociodemographic data from the patients was extracted from the hospital administrative database, while the economic analysis relies on observational data (hospital’s accounting department) and was estimated retrospectively. The study was carried out in accordance with the Helsinki Declaration [26]. Data was analyzed using a Google Drive Spreadsheet.

### The SJD Home Intervention

The intervention and characteristics of the families who used the service has been documented in previous studies [20]. When the care team, whether from the hospitalization ward, outpatient department, or emergency department, detects a potential case of hospitalization at home, it contacts the referent of this program, which evaluates it according to the inclusion criteria (30 minutes of isochronous, clinical stability, voluntary consent, and adequate living conditions in the home). The family is then informed about the home care service, and if they agree to participate, they are asked to give informed consent. Finally, the nurse of the team trains the family to be able to carry out the necessary care and delivers a kit. The program is thought to have a maximum of 12 patients; therefore, 15 kits are available. This kit contains the four devices for remote telemonitoring (thermometer, pulse oximeter, blood pressure monitor, and scale) and a tablet that uses Bluetooth with specific software that records device information and allows video calls. The service includes two types of health care: face-to-face, with a daily visit from a pediatrician or nurse, and 24-hour continuous care with real-time telemonitoring by nurses (between 8 AM to 10 PM) and by the emergency department staff (between 10 PM to 8 AM).

### Participants

From November 1, 2019, to June 30, 2020, a total of 357 patients received the pediatric tele–home care service. Among these episodes, only those who were first admitted to the hospital and subsequently were admitted to home hospitalization were selected. We detected three types of patients. First, some were admitted to the tele–home care program to end their treatment; these patients were fairly stable and had shorter stays. Second, some patients had pathologies that required a longer stay. Third, some patients had an underlying pathology. With the aim of having a more precise control group, we only included the first group of patients. The principal pathologies seen at home are acute respiratory diseases (bronchospasm, bronchiolitis, pneumonia), infections in need of intravenous treatment (eg, urinary infections, sepsis, skin and soft tissue infections, and otorrinolaringologic infections), nephrotic syndromes, and wounds in need of nurses’ healing. The main procedures done at home are oxygen therapy, nebulizations, and intravenous treatments (antibiotics and serum therapy). Although the main referral service is general pediatrics, other departments that also refer patients to the tele–home care program are surgery, nephrology, or oncology among others. The resulting study population included 181 patients.

A historical control group of patients with the same clinical casuistry and diagnostics to the children hospitalized at home were used for comparison. A review was made for diagnoses of comparable patients maintaining the same criteria of principal diagnostic, principal procedure, and service origin (pediatrics). All patients of the usual care that were used as a comparison group met all the inclusion criteria to be admitted to the program, except the 30 minutes of isochronous (children living further cannot be included in the treatment group for logistic reasons).

### Outcome Measures

Although in the usual care model personnel expenses include wages of pediatricians, nurses, residents, and nursing assistants, the tele–home care program is operated only by pediatricians and nurses. With respect to operating expenses, pharmacy, fungibles, and various purchase costs are included. Expenses per patient consist of the costs of the medicines given to patients. Laboratory, anatomy, diagnostic imaging, and blood bank costs are covered in the intermediate care costs. Intermediate noncare costs include the costs of admissions, stretcher bearers, cleaning of the spaces and clothing, menus offered to the hospital’s patients, and other intermediate expenses. Of these, the only ones attributable to the tele–home care program are laboratory, admissions, and blood bank costs. Some expenses are specific to the tele–home care program, such as the cost of the transportation, the renting of the tablet, and other purchases. Lastly, there are some structural expenses, such as the costs of supplies, amortizations of the computer system, and other expenses. Only the last two are included as tele–home care costs. The quantification of costs is done by the hospital’s own accounting department using administrative data. All costs are with prices for the year 2020. The study does not take into account any other amortization costs, as they are considered nonsignificant.

## Results

A total of 181 patients with ages between 0 and 21 (average 3.95, SD 5.00, median 2) years used the program. A total of 91 (50.3%) were female. The most frequent diagnoses were related to a respiratory disease (86/181, 47.5%), infection (51/181, 28.2%), and other less common pathologies. On average, patients spent 1.94 (SD 1.25) days at the hospital before being transferred to their homes, where they stayed for 2.82 (SD 1.25, min 1.10, max 8.38) days. This means that, in total, the mean of the whole hospitalization (conventional hospitalization plus home hospitalization) was 4.76 days. In comparison, the average total hospitalization of the control group was 3.73 (SD 2.47) days.

Table 1 shows the total average expenditure for a hospital and in-home hospitalization of a 24-hour stay, the difference between both to estimate the savings, and the percentage that each type of cost represented in the total amount of savings.

**Table 1:** Costs per day, according to type of hospitalization, by size of saving.

Type of cost	Usual care (US $)	Tele–home care (US $)	Variation (US $)	Total variation (%)^a^	Total savings (%)
Staff	261.51	158.67	102.83	33.52	35.50
Noncare intermediates	118.08	21.99	96.09	31.32	33.17
Structural	140.80	85.63	55.16	17.98	19.04
Intermediates	27.29	7.37	19.91	6.49	6.88
Operating	26.50	10.85	15.65	5.10	5.40
Tele–home care	N/A^b^	17.17	–17.17	5.60	N/A
Total	574.19	301.71	272.48	100.00	100.00

^a^In absolute terms.

^b^N/A: not applicable.

A 24-hour stay at the hospital costs US $574.19, while the in-home hospitalization costs US $301.71 per day, representing a saving of almost half (48%) of the cost compared to usual care. The main saving drivers were the personnel costs (US $102.83/US $289.66, 35.5% of the total), intermediate noncare costs (US $96.09/US $289.66, 33.17%), and structural costs (US $55.17/US $289.66, 19.04%), all of them accounting for 87.72% (US $254.09/US $289.66) of the total savings. The cost types are detailed in Table 2, which also shows that the only incremental expense between the two interventions was the operating cost of the home hospitalization program (mainly the professional’s travel costs and the devices used for telemonitoring).

**Table 2:** Costs per day, by type of hospitalization. Most important items (disaggregated).

Type of cost			Conventional care (US $)		Tele–home care (US $)		Difference (US $)		Savings (%)
**Personal**									
	Optional	83.92		65.76		18.16		6.66	
	Residents	21.45		N/A^a^		21.45		7.87	
	Nursery	116.46		92.91		23.55		8.64	
	Auxiliaries	39.67		N/A		39.67		14.56	
	Total staff	261.51		158.67		102.83		37.74	
**Intermediation care**									
	Admissions + secretariat	23.47		21.99		1.48		0.54	
	Bedding holders	16.35		N/A		16.35		6.00	
	Cleaning + laundry	25.57		N/A		25.57		9.38	
	Menu	40.01		N/A		40.01		14.69	
	Intermediate	12.66		N/A		12.66		4.65	
	Total intermediate noncare	118.08		21.99		96.09		35.27	
**Structure**									
	Informatics	8.41		8.41		N/A		0.00	
	Supplies/maintenance	53.69		N/A		53.69		19.71	
	Structural	77.22		77.22		N/A		0.00	
	Depreciation	1.47		N/A		1.47		0.54	
	Total structure	140.80		85.63		55.16		20.25	

^a^N/A: not applicable.

In relation to staff costs, the results shown are lower for all types of professionals. The main savings are due to the absence of auxiliary staff (US $39.67/US $272.48, 14.56% of the total). As for other professionals, the costs are lower due to the lower ratio of professionals per patient. Regarding the intermediate noncare expenses, the main savings are given by the costs of food (US $40.01/US $272.48, 14.69%), cleaning and laundry (US $25.57/US $272.48, 9.38%), and bedding (US $16.36/US $272.48, 6%). Finally, in terms of structure, most savings were given by supply costs (US $53.69/US $272.48, 19.71%).

Table 3 summarizes the costs by discharge, weighting the daily cost of each type of stay by its average duration. Home hospitalization involves a total stay 27.61% longer but at a daily cost of almost half; it represents a saving of US $176.70 (9.01%) per stay.

**Table 3:** Cost per discharge.

	Hospital stay (days), mean	Home stay (days), mean	Total stay (days)	Total cost (US $)
Home	1.94	2.82	4.76	1964.76
Hospital	3.73	0	3.73	2141.75

## Discussion

### Principal Findings

This study is an economic analysis from a hospital’s perspective that compares the costs of two competing treatments. On the one hand, home hospitalization allows the release of hospital beds occupied by patients who, due to their clinical situation, can stay at home. This space is especially needed in the winter because there are peaks in demand motivated by the high incidence of respiratory viruses. Thus, this intervention represents a *de facto* expansion of the hospital bed capacity. On the other hand, at times, with few patients hospitalized at home, the flexibility in human resource management and the ability of professionals to carry out their work in other services or areas of the hospital would minimize their opportunity cost. With these conditions, home hospitalization would be an efficient option thanks to the abundance of variable costs associated with this model. This is consistent with a recent study centered in telemedicine in pediatrics that emphasizes that patients, health care professionals, and caregivers may benefit from using both telemedicine services and traditional, in-person health care services [27].

In terms of safety, some articles show that it appears that hospital at home is a safe and acceptable form of care [28]. Additionally, some studies demonstrate that clinical effectiveness of both services was not significantly different: children presenting common pathologies that require hospital treatment but not its infrastructure could be managed at home with similar outcome measures to traditional hospital care [11]. For example, a recent systematic review that focuses on malignant and nonmalignant hematology concluded telemedicine provides similar or improved health care compared to face-to-face encounters in both pediatric and adult populations [29]. The readmission rate for home care was not significantly higher than for hospital care [20]. Additionally, in terms of satisfaction, a British study shows that 90% of parents and 63% of children stated a clear preference for home hospitalization, citing less psychosocial disruption and a perception that children recover more quickly with comfortable surroundings [11].

The facilities offered by digital health tools, combined with a gradual decline in the cost of gadgets to comparatively insignificant levels, open the door to a set of possibilities for cost-effective interventions in the field of health. The result of this work fits in with other studies that point to the positive economic impacts of telemedicine [30,31]. In the context of COVID-19, these possibilities make even more sense insofar as they can reduce travel, social contact, and consequently intrahospital infections [32]. Recent studies claim that digital approaches have played and will play substantial roles as invaluable and reliable resources to overcome restrictions and challenges imposed during the COVID-19 pandemic and to increase access to effective, accessible, and consumer-friendly care to more pediatric patients and families [33]. For example, another recent paper states that despite its limitations, the expansion of digital health care due to the COVID-19 pandemic is likely to have equitably increased access to health care for many families, especially those living rurally and with limited financial means. It is also likely to have reduced the anxiety experienced by some children in medical settings and allowed health professionals to gain a better understanding of their patients’ living circumstances [34].

This analysis has several limitations. First, this study spans from November 1, 2019, to June 30, 2020. This includes some important times for the Spanish health system due to COVID-19, and special measures had to be implemented: COVID-19 patients used the program, and although the hospital is pediatric, it accommodated adult patients. Hospital occupancy declined due to the low incident of COVID-19 and other pathologies on children in this period. Second, this analysis only includes one typology of patients: the ones who were admitted to the tele–home care program to end their treatment. It would be interesting to include the other patients in future studies. Third, provider’s perspective does not include aspects that go beyond their interests, such as the possible cost of caring for the child at home by families (loss of productivity, material costs). Further research should enlarge the focus of the study and include and broaden all the potential effects of in-home hospitalization.

### Conclusion

Our analysis shows that pediatric tele–home care is 9% less expensive compared to regular hospital care while offering a quality service preferred for the children and their families, and that emptied beds for more complex cases. The use of telemedicine in the pediatric setting may serve for improving provider efficiency, lowering health system costs, and achieving greater patient satisfaction [18]. These results motivate the most widespread implementation of the pediatric tele–home care.

## Abbreviations

CHEERS: Consolidated Health Economic Evaluation Reporting Standards
